# Dopamine and dopamine receptor D1 as a novel favourable biomarker for hepatocellular carcinoma

**DOI:** 10.1186/s12935-021-02298-9

**Published:** 2021-10-30

**Authors:** Zhihui Wang, Peihao Wen, Bowen Hu, Shengli Cao, Xiaoyi Shi, Wenzhi Guo, Shuijun Zhang

**Affiliations:** 1 Department of Hepatobiliary and Pancreatic Surgery, The First Affiliated Hospital of Zhengzhou University, No. 1 Jianshe Road, Zhengzhou, 450052 Henan China; 2 Zhengzhou Key Laboratory of Hepatobiliary & Pancreatic Surgery and Digestive Organ Transplantation at Henan Universities, Zhengzhou, 450052 China; 3 Open and Key Laboratory of Hepatobiliary & Pancreatic Surgery and Digestive Organ Transplantation at Henan Universities, Zhengzhou, 450052 China; 4 Henan Key Laboratory of Digestive Organ Transplantation, Zhengzhou, 450052 China

**Keywords:** Hepatocellular carcinoma, DRD1, Prognosis, Immune infiltration, Coexpressed genes

## Abstract

**Background:**

Hepatocellular carcinoma (HCC) remains one of the most common malignant tumours worldwide. Therefore, the identification and development of sensitivity- genes as novel diagnostic markers and effective therapeutic targets is urgently needed. Dopamine and dopamine receptor D1 (DRD1) are reported to be involved in the progression of various cancers. However, the crucial role of DRD1 in HCC malignant activities remains unclear.

**Methods:**

We enrolled 371 patients with liver hepatocellular carcinoma (LIHC) from The Cancer Genome Atlas (TCGA) to detect the expression and functions of DRD1. The Tumour Immune Estimation Resource (TIMER), UALCAN database, Kaplan–Meier plotter, cBioPortal database, and LinkedOmics database were utilized for the systematic investigation of DRD1 expression and related clinical features, coexpressed genes, functional pathways, mutations, and immune infiltrates in HCC.

**Results:**

In this study, we determined that DRD1 expression was decreased in HCC tumour tissues versus normal tissues and that low DRD1 expression indicated a poor prognosis. The significance of DRD1 expression varied among different tumour samples. The somatic mutation frequency of DRD1 in the LIHC cohort was 0.3%. The biological functions of DRD1 were detected and validated, and DRD1 was shown to be involved in various functional activities, including metabolism, oxidation, mitochondrial matrix-related processes and other related signaling pathways. In addition, out study indicated that DRD1 had significant correlations with the infiltration of macrophages, B cells and CD+ T cells in HCC.

**Conclusions:**

These findings demonstrated the rationality of the potential application of DRD1 function as a novel biomarker for HCC diagnosis and a therapeutic target for HCC treatment.

**Supplementary Information:**

The online version contains supplementary material available at 10.1186/s12935-021-02298-9.

## Background

Hepatocellular carcinoma (HCC) is globally ranked fifth among all cancers in terms of incidence and second in terms of cancer-related mortality in males [1]. Although great improvement has been made in HCC diagnosis and treatment, systemic treatments for liver cancer are still limited, especially for advanced stage disease, due to its high rate of metastatic spread and recurrence [2]. The low 5-year overall survival rate of advanced HCC patients (< 5%) has been attributed to a poor prognosis, owing to the high incidence and mortality rates and limited treatment options [3]. Therefore, earlier detection of HCC is of great importance to decrease the mortality rate and to provide therapeutic targets for early-stage HCC [4]. The identification of high sensitivity-conferring genes for HCC as effective targets might provide novel insights into the aetiology of HCC. In addition, further in-depth studies of HCC biomarkers may help to achieve better prognosis prediction and to guide tailored therapy, which is of great practical significance. Over the years, many studies on HCC have examined the mechanisms underlying HCC biomarker identification. In recent years, growing evidence has demonstrated that the dopamine receptor gene family plays an essential role in tumour initiation and progression [5, 6]. Dopamine and dopamine receptor D1 (DRD1), a key member of the dopamine receptor family, exert positive effects on lung cancer [7], pancreatic cancer [8], and glioblastoma [9]. DRD1 is a member of the seven transmembrane domain trimeric guanosine 5'-triphosphate (GTP)-binding protein-coupled receptor family, which also includes the D2, D3, D4 and D5 receptors [10]. These 5 receptors are divided into 2 subgroups, namely, D1-like receptors (DRD1 and DRD5) and D2-like receptors (DRD2, DRD3 and DRD4) [11]. The two clusters show different biochemical and pharmacological signatures [11]. Accumulating evidence has demonstrated that DRD1 plays vital roles in immune regulation, hormone secretion mediation, gene expression, metabolic profile regulation [12], stress-induced behaviours [13], addiction-like behaviours [14], and so on. Aberrant DRD1 expression has been validated to correlate with various tumours and to be involved in tumour cell migration [15], tissue inflammation [10], autophagy activation [16], tumour growth [6], tumour progression and cancer cell biology [5].

Studies have demonstrated that dopamine exerts antitumour effects through DRD1-mediated Lrp5-CCN4 inhibition in brain tumours [17]. Research on glioblastoma has shown that DRD1 expression is closely and positively associated with a favourable clinical prognosis. In addition, DRD1 activation was validated to suppress glioblastoma tumour cell progression [9]. It was also established that DRD1 activation might enhance antitumour activities in pancreatic cancer [8]. Our previous study determined that DRD1 acts as a key regulatory gene in HCC immune signature identification [18]. However, the specific mechanism by which DRD1 regulates HCC proliferation and migration is still unclear.

In this study, we aimed to explore DRD1 mRNA expression and clinical signatures based on public databases to investigate its coexpressed genes and closely related pathways and to validate DRD1 activities in clinical samples and in vitro studies.

## Methods

### Ethical approval for HCC human samples

Eight paired tumour tissues and adjacent normal tissues from HCC patients were obtained from The First Affiliated Hospital of Zhengzhou University (Zhengzhou, China) from 2018 to 2020, and all patients signed informed consent forms. This study was approved by the Ethical Committee of The First Affiliated Hospital of Zhengzhou University.

### The inclusion criteria for HCC samples

The preoperative diagnosis of HCC was based on the criteria of the American Association for the Study of Liver Diseases [19]. In addition, tissue samples were obtained during surgery, and clinical samples were sent to the Department of Pathology of The First Affiliated Hospital of Zhengzhou University. These samples were diagnosed as HCC by at least two senior pathologists. These HCC patients had not received antitumour therapy before diagnosis.

### The exclusion criteria for HCC samples

There were 4 criteria for exclusion from the study. These were (1) the precedent of other tumours, (2) lack of regular follow-up, (3) incomplete clinical data, and (4) life expectancy of less than 3 months.

### RNA extraction and qPCR

Total RNA was extracted from HCC tumour tissues and normal tissues using an RNA Extraction Kit (Qiagen, Germany) according to the manufacturer’s protocol. The RNA concentration of total RNA was measured by a NanoDrop spectrometer (Thermo Fisher Scientific). The RNA purity (optical density) of each sample was larger than 2.0. After that, RNA was reverse transcribed to obtain complementary deoxyribose nucleic acids (cDNAs) with PrimeScript RT Reagent (Vazyme Biotech Co. Ltd. Nanjing). The qRT-PCR protocol was conducted using Vazyme ChamQ SYBR qPCR Master Mix obtained from Vazyme Biotech Co. Ltd. (Nanjing, China). PCR was carried out as follows: initial denaturation at 95 °C for 30 s, followed by 40 cycles of 10 s at 95 °C and 30 s min at 60 °C, and a final extension at 95 °C for 15 s, 60 °C for 60 s, and 95 °C for 15 s (LightCycler Strep-A assay (Roche Applied Science, Indianapolis, Ind)). The mRNA expression levels of the target genes were quantified using the 2-ΔΔCq method [20] and normalized to GAPDH mRNA expression levels. The following primers were used: GAPDH F, 5’-TGCACCACCAACTGCTTAGC-3’ and R, 5’-GGCATGCACTGTGGTCATGAG-3’; DRD1 F, 5’-TGGCATGTGAAGCTCCTCTC-3’ and R, 5’-CCTGTCGATTGTCAGCAGGATTA-3’.

### Tumour Immune Estimation Resource (TIMER)

The results from TIMER (available at http://cistrome.org/) [21], a comprehensive resource for the systematic analysis of immune cell infiltrations across 33 types of cancer types, were validated using pathological estimations. We applied the TIMER database and reviewed DRD1 mRNA expression in various cancers. Herein, we also investigated the relationship between the transcriptional expression level of DRD1 and associated tumour-infiltrating immune cells, such as T cells, B cells, macrophages, dendritic cells (DCs), and natural killer cells (NK). TIMER was also applied to estimate the immune purity, immune cell infiltration level and DRD1 expression in multiple cancers.

### UALCAN database analysis

UALCAN (http://ualcan.path.uab.edu/) [22] is a bioinformatics tool for analysing and visualizing data from The Cancer Genome Atlas (TCGA). We screened the UALCAN database to investigate DRD1 relative transcriptional expression in HCC tumour tissues compared with adjacent normal tissues. In addition, mRNA expression and related clinical characteristics, such as race, tumour grade, sex, tumour node and metastasis (TNM) stages, were considered.

### *Survival analysis *via* Kaplan–Meier plotter*

Kaplan–Meier plotter is an online database (www.kmplot.com) with data sources such as the Gene Expression Omnibus (GEO), European Genome-phenome Archive (EGA), and TCGA. We adopted Kaplan–Meier analysis to estimate the prognostic relevance of DRD1 expression levels in the TCGA cohort.

### cBioPortal database analysis

To investigate the genetic mutations of DRD1 in HCC and the correlations with prognosis evaluations, we performed cBioPortal analysis. cBioPortal (https://www.cbioportal.org) [23] provides a platform for the interactive exploration of multidimensional cancer genomic datasets. We used this database to explore DRD1 transcriptional expression and its correlated mutations. In our analysis, the genomic profiles of DRD1 included mutations and putative copy number alterations. Genetic mutations in DRD1 and its correlated prognostic indicators, such as overall survival (OS) and disease-free survival (DFS), were analysed with Kaplan–Meier plots.

### LinkedOmics database analysis

LinkedOmics (http://www.linkedomics.org/login.php) [24] is a web-based online platform for analysing multiomics data within and across 32 TCGA cancer‐associated multidimensional datasets. In this study, LinkedOmics was performed to investigate the correlation between DRD1 expression and other coexpressed genes. The protein–protein interactions (PPIs) were determined, and the PPI network was constructed by means of the cBioPortal database.

## Results

### DRD1 transcriptional expression in pan-cancers and the clinical features of the HCC cohort

**Fig. 1:**
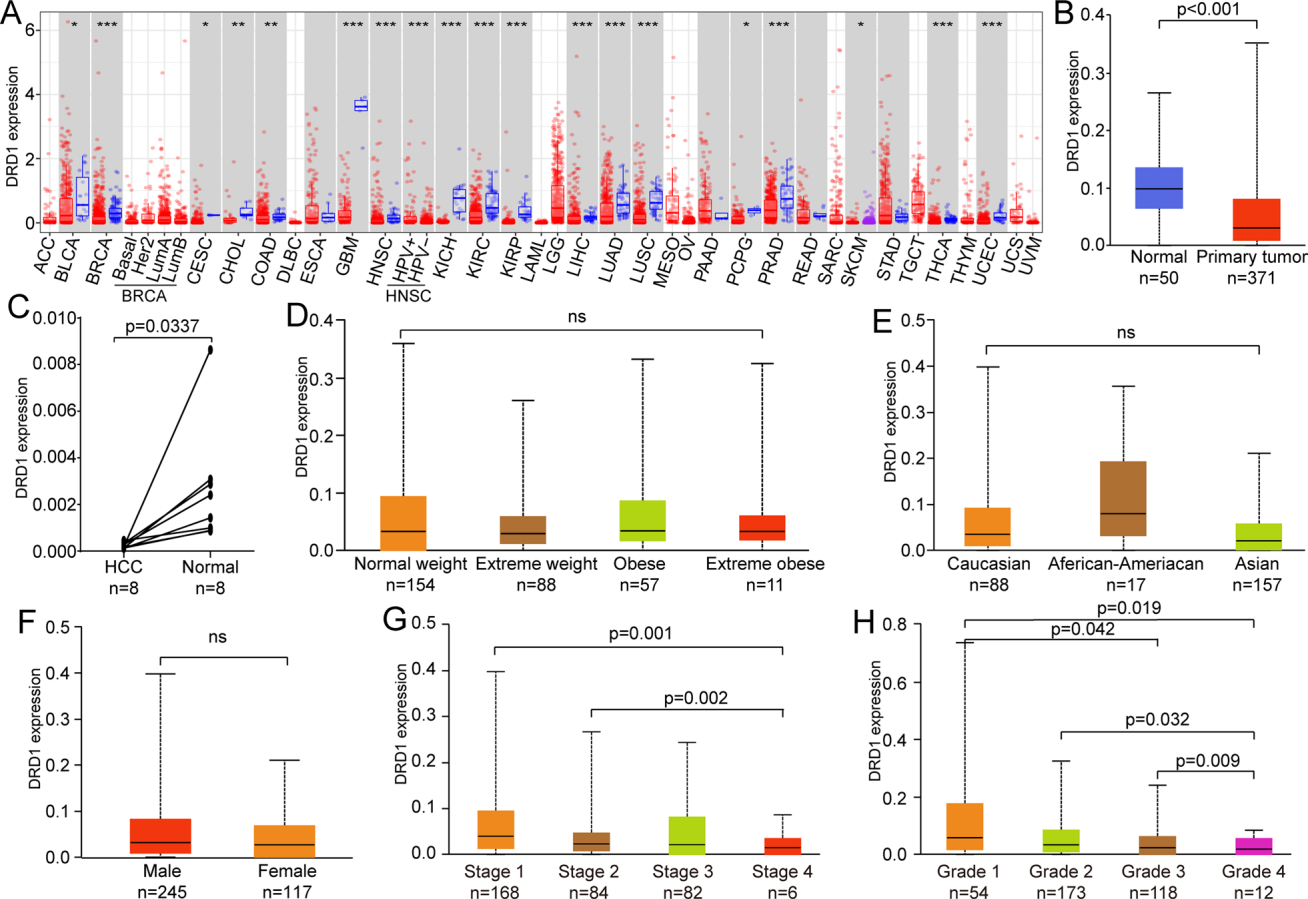
DRD1 expression level and associated clinical characteristics in HCC. **a** DRD1 mRNA expression levels across cancers. **b** The DRD1 mRNA expression level was significantly decreased in primary tumour tissues compared with normal tissues. **c** The DRD1 mRNA expression level was decreased in HCC tissue compared with normal tissue. **d**–**f** There was no significant difference in DRD1 mRNA expression levels between patients grouped by weight, tumour grade, and sex. **g**, **h** The difference in DRD1 mRNA expression levels between patients with different tumour stages and tumour grades

We used TIMER to systematically investigate the mRNA expression level of DRD1 among 33 cancer types and determined that DRD1 was significantly decreased in bladder urothelial carcinoma (BLCA), breast invasive carcinoma (BRCA), cervical squamous cell carcinoma and endocervical adenocarcinoma (CESC), cholangiocarcinoma (CHOL), colon adenocarcinoma (COAD), glioblastoma multiforme (GBM), head and neck squamous cell carcinoma (HNSC), kidney chromophobe (KICH), kidney renal papillary cell carcinoma (KIRC), kidney renal papillary cell carcinoma (KIRP), liver hepatocellular carcinoma (LIHC), lung adenocarcinoma (LUAD), lung squamous cell carcinoma (LUSC), pheochromocytoma and paraganglioma (PCPG), prostate adenocarcinoma (PRAD), skin cutaneous melanoma (SKCM), thyroid carcinoma (THCA), and uterine corpus endometrial carcinoma (UCEC) (Fig. 1a). Our previous studies have identified DRD1 as a key hub gene in the immune-phenotype gene expression regulatory network of HCC [18]. Therefore, we deeply explored DRD1 expression, which was remarkably decreased in HCC primary tumour tissues compared with normal tissues (Fig. 1b). The PCR results of eight pairs of tumour tissues and adjacent normal tissues indicated that DRD1 expression was decreased in HCC tissues (p = 0.0337, Fig. 1c). In the LIHC cohort, DRD1 expression showed no significant variation with race, sex, or patient weight (Fig. 1d–f). Interestingly, DRD1 expression showed obvious differences between patients with different TNM stages and tumour grades. DRD1 had significantly higher expression in the early-stage (stage 1 and stage 2) HCC cohort and in the normal cohort compared with the advanced-stage (stage 4) cohort (Fig. 1g). Moreover, DRD1 expression in grade 4 HCC was lower than that in grade 1, 2 and 3 HCC (Fig. 1h).

### Low DRD1 expression was associated with a poor prognosis

**Fig. 2:**
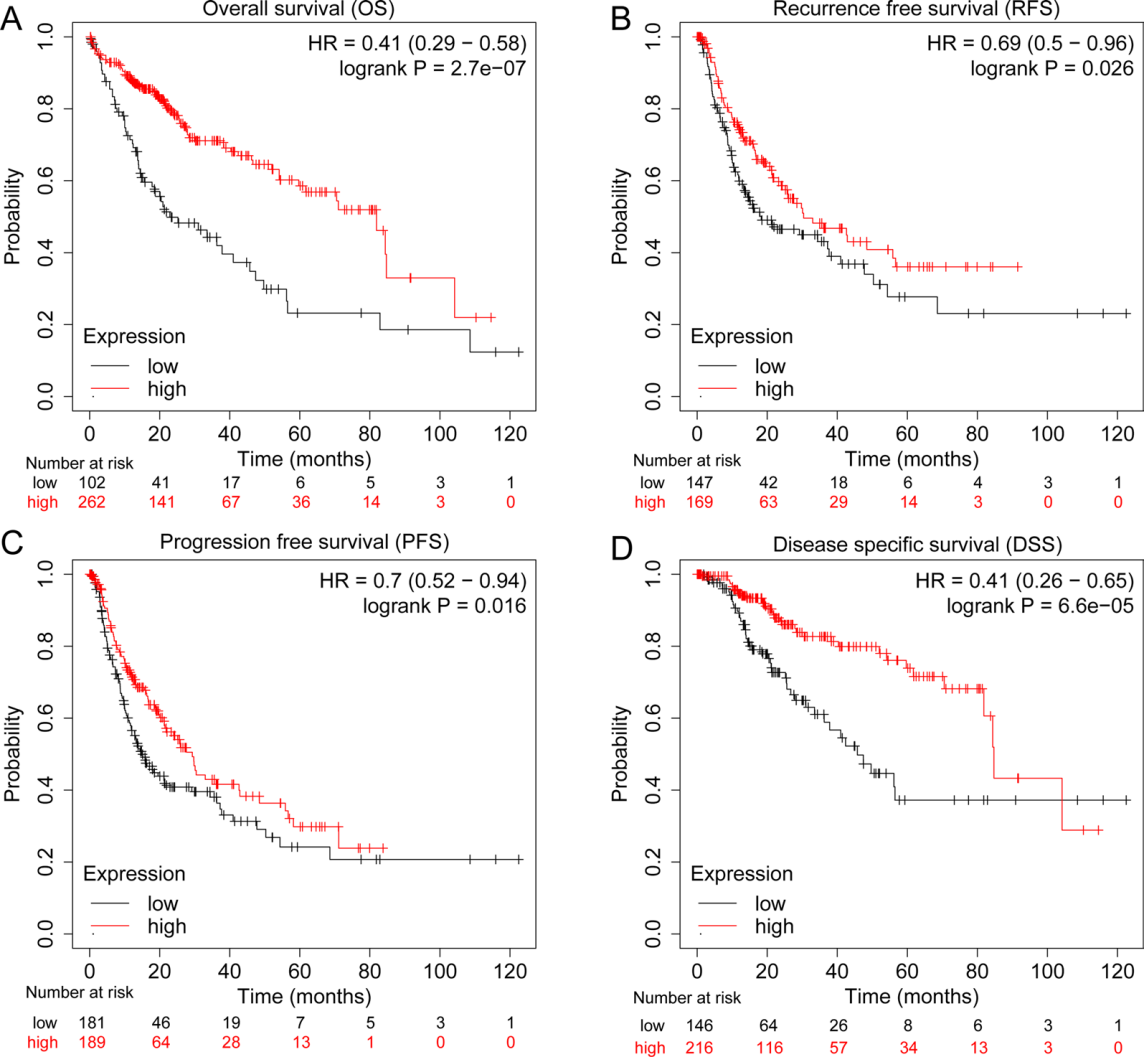
Differences in clinical prognosis between the low and high DRD1 expression groups. **a** OS analysis indicated that low DRD1 expression indicates a poor prognosis. **b** The RFS curve indicated that low DRD1 expression is associated with shorter RFS time. **c** The high DRD1 expression group had better PFS than the low expression group. **d** The high DRD1 expression group had better DSS than the low expression group

To estimate the impact of decreased DRD1 expression on the associated clinical prognosis, we evaluated the effect of low DRD1 expression on progression-free survival (PFS), overall survival (OS) and disease-specific survival (DSS). The results indicated that low DRD1 expression was significantly associated with poor survival in terms of DSS, DFS, recurrence-free survival (RFS) and PFS, in the TCGA-LIHC dataset, as shown in Fig. 2a–d.

### Mutations of DRD1 in the LIHC cohort

**Fig. 3:**
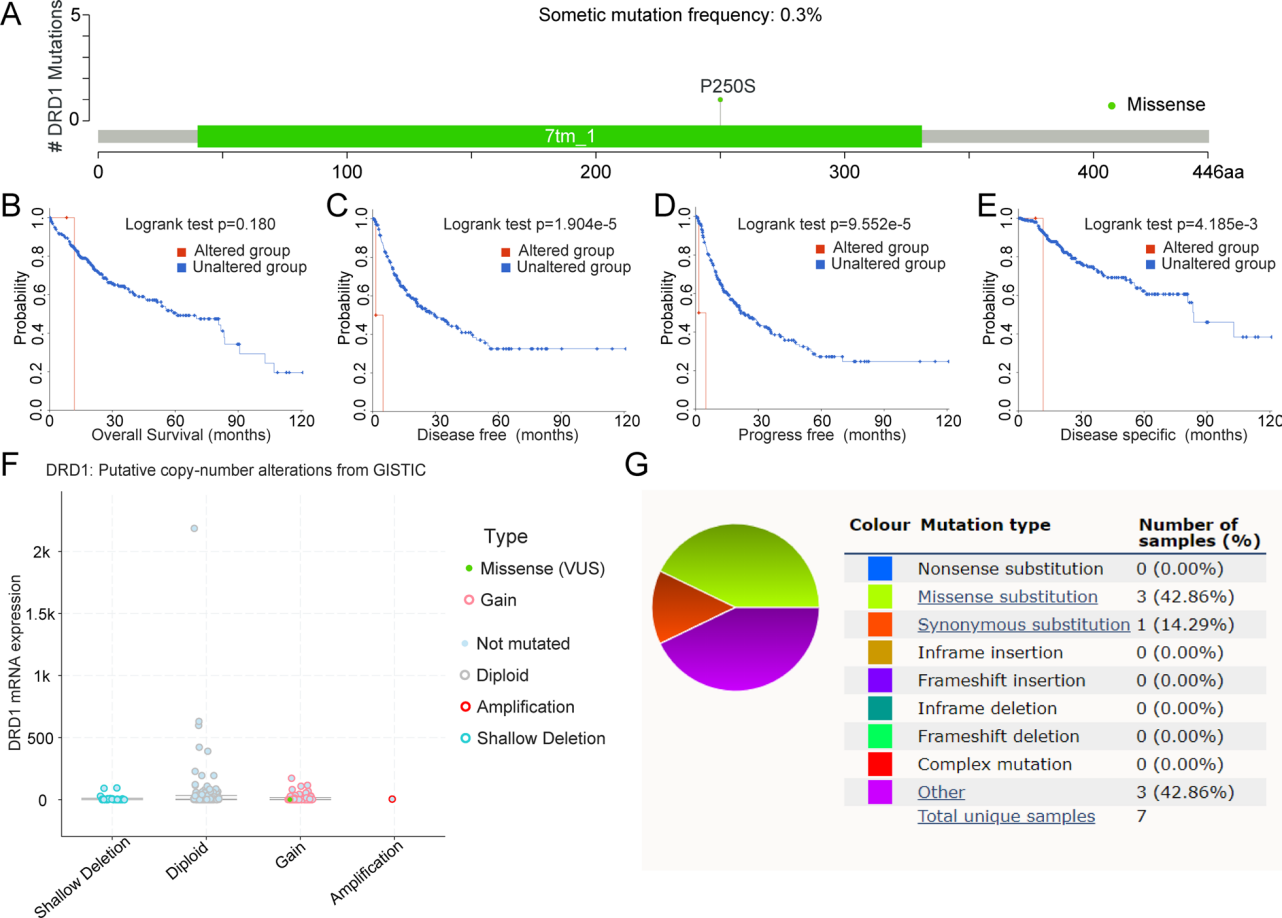
DRD1-associated genomic mutations in HCC. **a** DRD1-associated somatic mutations. **b**–**e** The differences in clinical prognosis between the mutation group and the wild-type in terms of OS, DFS, PFS, and DSS. **f** DRD1 mRNA expression between groups with different mutation types. **g** DRD1-associated mutation types in HCC

To comprehensively describe DRD1-related gene alterations, we performed cBioPortal database analysis and determined that the somatic mutation frequency was 0.3%. There was only 1 case of missense mutation (P250S) (Fig. 3a). The mutant versus wild-type DRD1 subgroups exhibited no significant difference in OS (Fig. 3b). The DFS analysis indicated that the wild-type DRD1 subgroup had a better prognosis than the subgroup with DRD1 mutation (p = 1.904e−5, Fig. 3c). DSS analysis showed that the group with mutated DRD1 had a shorter survival time (p = 4.185e−3, Fig. 3d). The PFS results indicated that the wild-type DRD1 group had a longer predicted survival time (p = 9.552e−5, Fig. 3e). As shown in Fig. 3f**,** the DRD1-associated putative copy number alterations were shallow deletion, diploid, gain, and amplification. In this study, we observed variable gene copy numbers of DRD1 (defined as deep deletion, shallow deletion, diploid, gain or amplification) that significantly correlated with DRD1 mRNA levels. To further investigate the mutation distribution, we investigated the existence of various mutation types, including missense substitutions (42.86%), synonymous substitutions (14.29%), and others (42.86%). Each of the mutation types (A>G, C>T, C>G, G>A) accounted for 25% of all mutations (Fig. 3g).

### DRD1-related coexpressed genes in LIHC

**Fig. 4:**
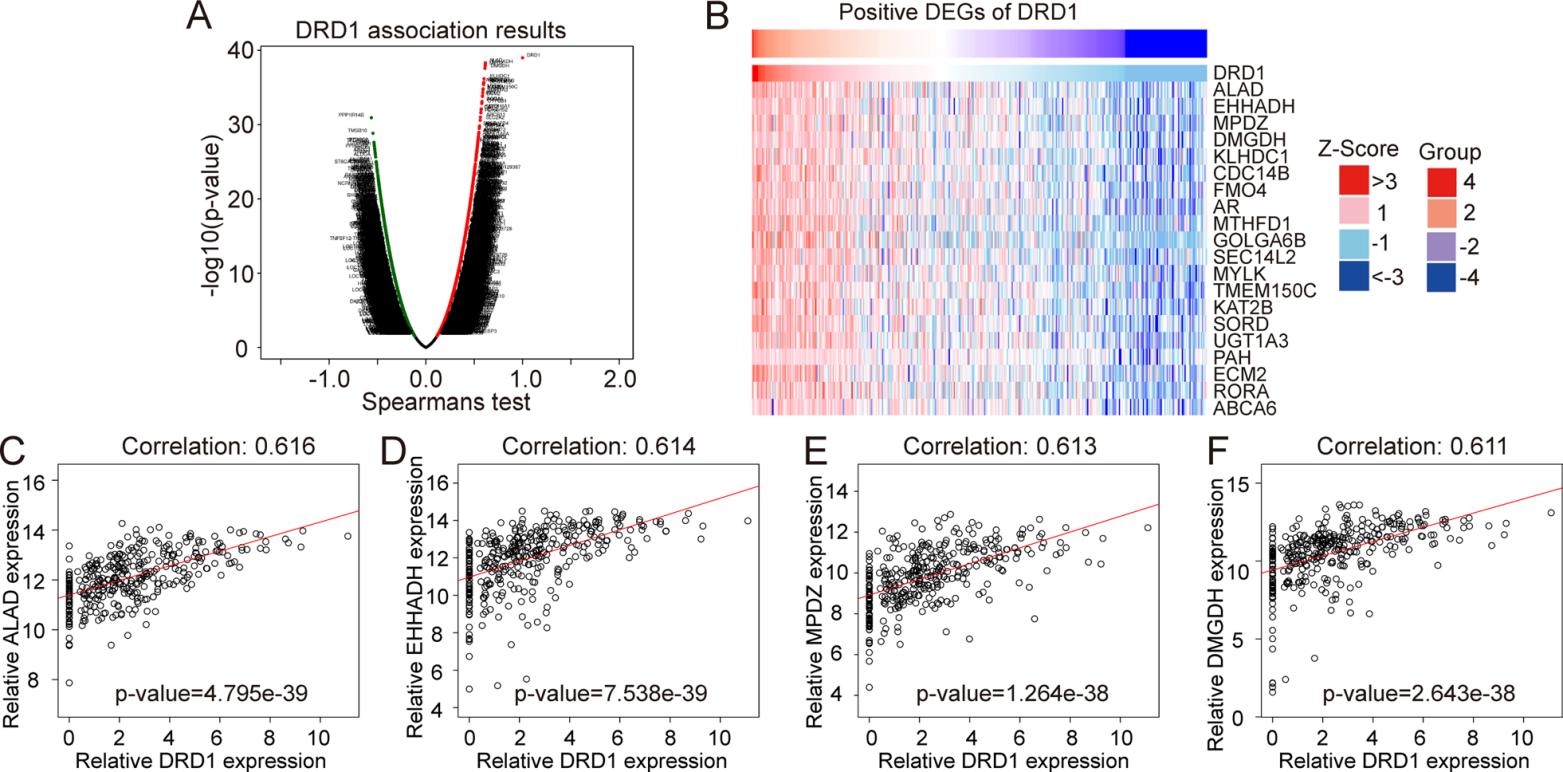
DRD1-associated differentially expressed genes and their correlations. **a** Differentially expressed genes positively and negatively associated with DRD1. **b** The top 20 differentially expressed genes positively associated with DRD1. The red column represents differentially expressed genes positively associated with DRD1. Blue represents the differentially expressed genes negatively associated with DRD1. **c**–**f** The correlation between the relative expression of DRD1 and that of ALAD, EHHADH, MPDZ, and DMGDH

The LinkedOmics database was adopted to further investigate the DRD1-associated coexpressed genes, and the results demonstrated that there were almost 222 genes in black dots, which represented a positive connection with DRD1 expression in the LIHC cohort, and there were 333 genes with negative correlations with DRD1 (Fig. 4a). We further plotted the top 20 expressed genes that were positively correlated with DRD1 by a heatmap (Fig. 4b). The correlation curves showed that the top four DRD1-related genes were ALAD (cor: 0.616, p = 4.795e-39), EHHADH (cor = 0.614, p = 7.538e−39), MPDZ (cor = 0.613, p = 1.264e−38), and DMGDH (cor = 0.611, p = 2.643e−38) (Fig. 4c–f).

### DRD1-related PPI network construction and identification of hub genes

**Fig. 5:**
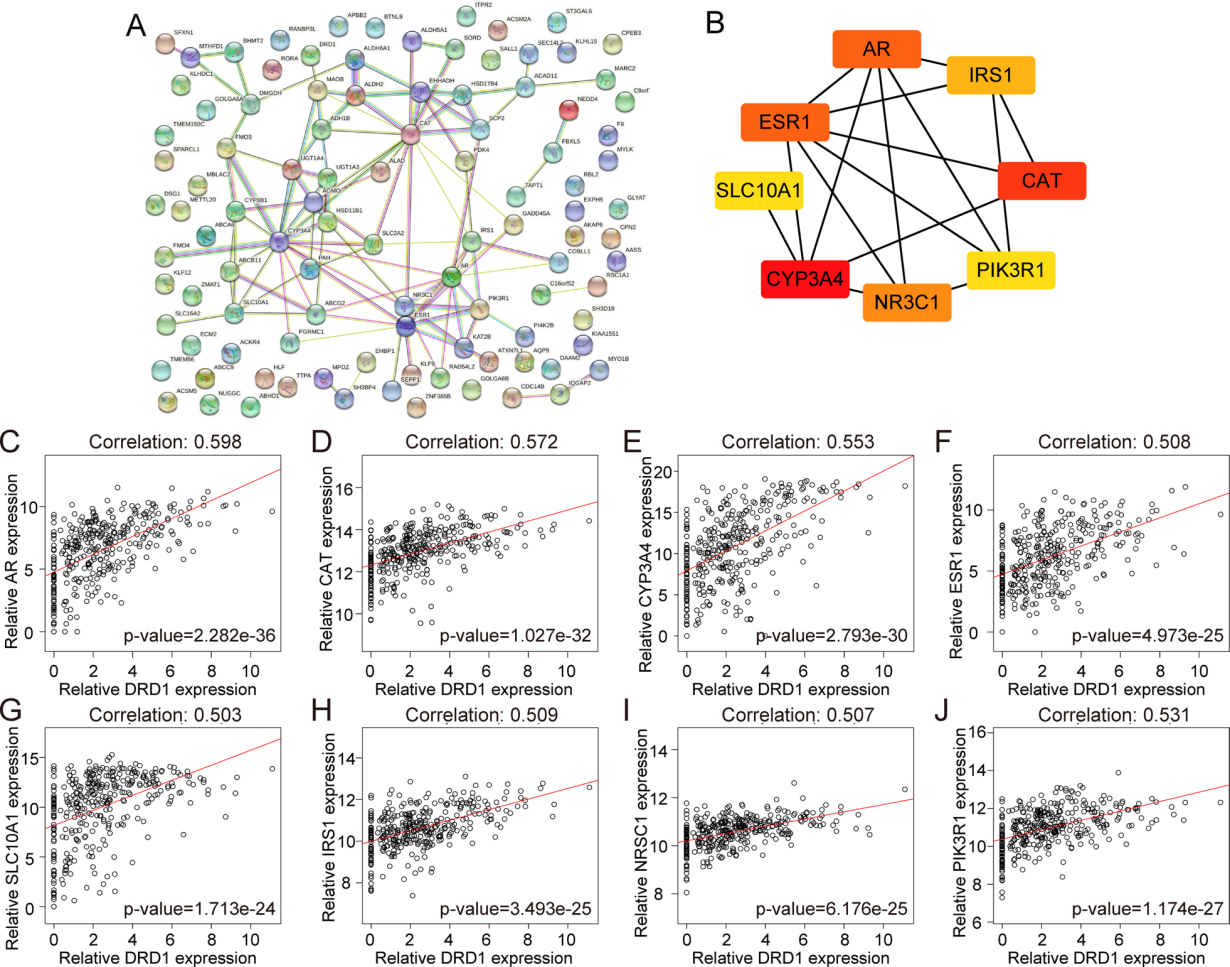
PPI network construction and identification of hub genes. **d** The PPI network of DRD1 in HCC. **b** Eight hub genes of DRD1 in the HCC cohort. **c**–**j** Correlation analysis of the expression of DRD1 and that of the 8 hub genes (AR, CAT, CYP3A4, ESR1, SLC10A1, IRS1, NRSC1, and PIK3R1)

We selected the top 100 coexpressed genes to build a PPI network with the LinkedOmics database (Fig. 5a). Furthermore, we identified the 8 hub genes with the highest degree scores. We further comprehensively analysed the correlation between hub genes and DRD1 expression. Based on the results, the genes were ranked by correlation coefficients as follows: AR (correlation: 0.598, p = 2.282e−36), CAT (correlation: 0.572, p = 1.027e−32), CYP3A4 (correlation: 0.553, p = 2.793e−30), ESR1 (correlation: 0.508, p = 4.793e−25), SLC10A1 (correlation: 0.503, p = 1.713e−24), IRS1 (correlation: 0.509, p = 3.493e−25), NRSC1 (correlation: 0.507, p = 6.176e−25), and PIK3R1 (correlation: 0.531, p = 1.174e−27) (Fig. 5b–j).

Furthermore, we detected the expression of hub genes. The expression levels of AR, CAT, CYP3A4, ESR1 and SLC10A1 were markedly upregulated in primary tumour tissues compared with normal tissues (Additional file 1: Figure S1a–e). However, the expression levels of IRS1 and NS3C1 were significantly downregulated in primary tumour tissues (Additional file 1: Figure S1f and g). Moreover, the expression levels of PIK3R1 mRNA showed no significant difference between tumour tissues and normal liver tissues (Additional file 1: Figure S1h). We adopted Kaplan–Meier analysis to assess the association between hub gene expression and the prognosis of patients with HCC. The results showed that patients with high AR expression had shorter overall survival times than those with low AR expression (Additional file 2: Figure S2a). Similar results were observed for CAT, CYP3A4, ESR1, SLC10A1, IRS1, NS3C1 and PIK3R1 (Additional file 2: Figure S2b–h).

### Pathway enrichment analysis

**Fig. 6:**
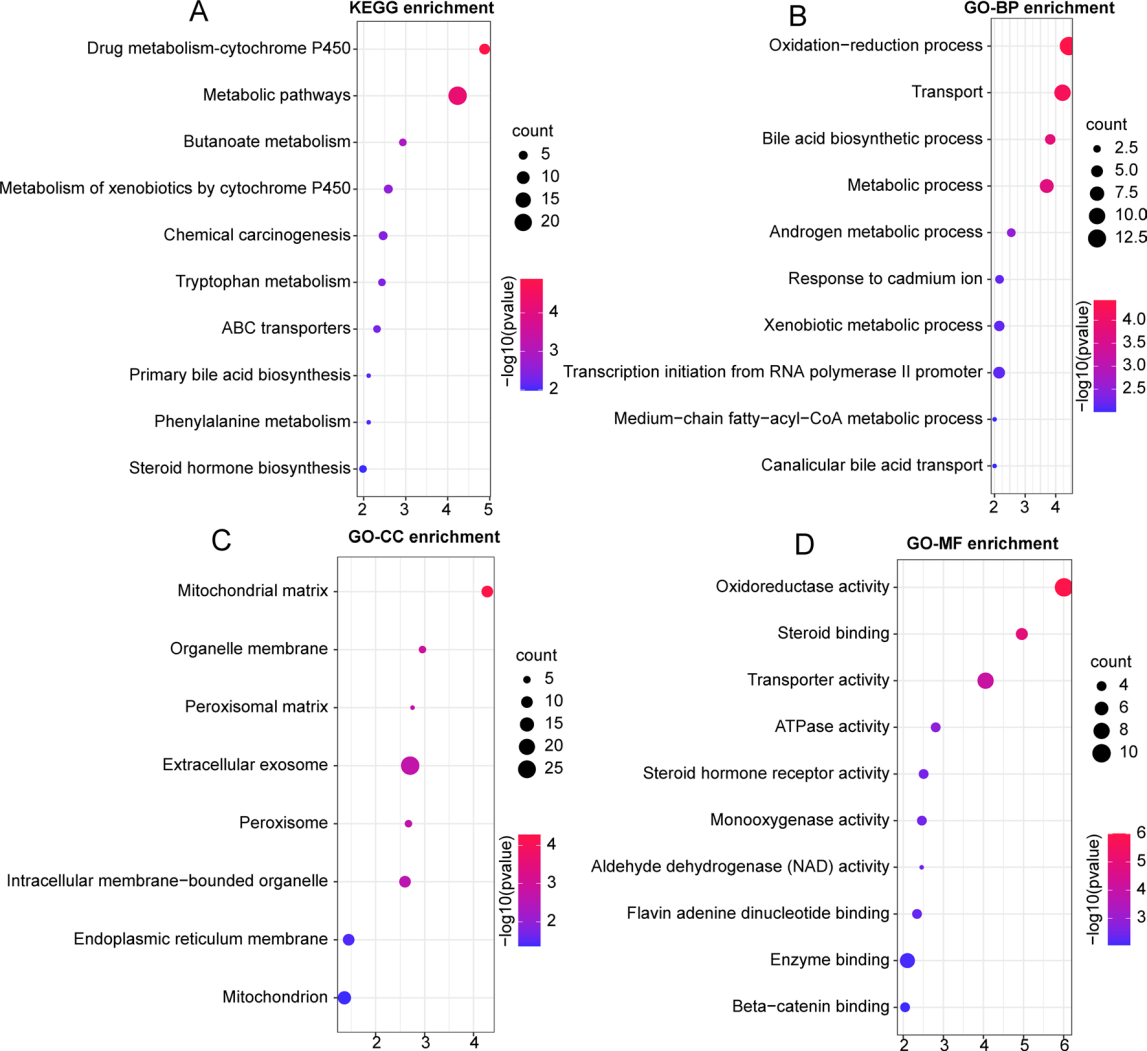
Functional enrichment analysis of DRD1-associated pathways. **a** KEGG functional analysis indicated that DRD1 had significant correlations with certain metabolic pathways. **b** GO-BP analysis showed that DRD1 was mainly enriched in the ‘oxidation–reduction process’ and ‘transport pathways’. **c** GO-CC analysis showed that DRD1 was mainly enriched in the ‘mitochondrial matrix’ and ‘extracellular exosome’. **d** GO-MF analysis demonstrated that DRD1 was mainly enriched in ‘oxidoreductase activity, ‘steroid binding’, and ‘transporter activity’

We applied LinkedOmics to further explore the functional role of DRD1 expression and its correlated genes. The KEGG analysis indicated the metabolic pathways where the DRD1-related coexpressed genes in LIHC were mainly located (Fig. 6a). Gene Ontology (GO) analysis covered enriched terms in three domains: biological process (BP), cellular component (CC), and molecular function (MF). The main enriched GO-BP terms were ‘oxidation–reduction process’, ‘transport’, ‘metabolic process’ and ‘bile acid biosynthetic process’ (Fig. 6b). The main enriched GO-CC terms were ‘cellular exosome’ and ‘mitochondrial matrix’ (Fig. 6c). The main enriched GO-MF terms were ‘oxidoreductase activity’, ‘steroid binding’, and ‘transporter activity’ (Fig. 6d).

### Estimation of immune cell infiltrations and immune purity

**Fig. 7:**
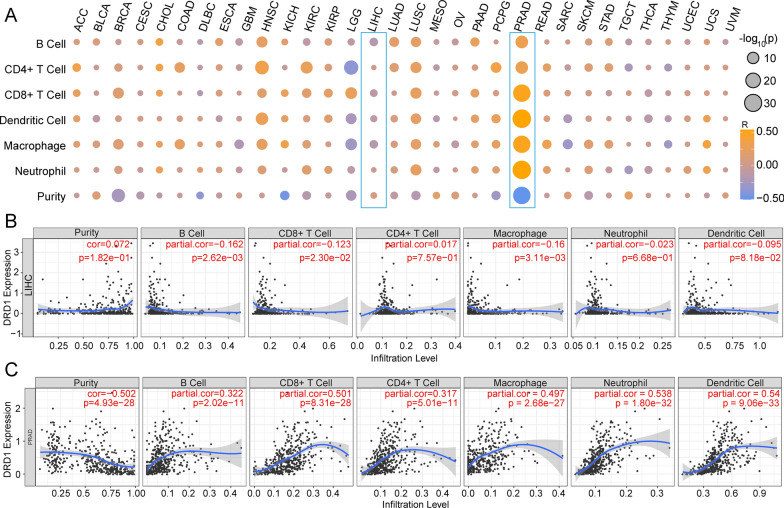
Immune landscape of DRD1 expression and immune cell infiltration. **a** Correlations between DRD1 expression and immune cell infiltration across cancers. **b** The correlations between DRD1 expression and immune purity/immune cell infiltration in the LIHC cohort. **c** The correlations between DRD1 expression and immune purity/immune cell infiltration in the PRAD cohort

We applied TIMER to highlight the DRD1 expression characteristics of immune cell infiltration and immune purity and determined the levels of immune cells such B cells, CD4+ T cells, CD8+ T cells, dendritic cells, macrophages, and neutrophils in a pancancer dataset. DRD1 expression across cancers and associated immune cell expression levels are shown in Fig. 7a. It was demonstrated that immune cells had a low correlation with DRD1 expression in LIHC; however, this correlation was significant in PRAD. Specifically, DRD1 expression in LIHC had a relatively weak correlation with immune purity (cor = 0.072, p = 1.82e−1) and immune cell infiltration (Fig. 7b). In PRAD, the correlation between DRD1 expression and immune purity was highly significant (correlation = 0.502, p = 4.93e−28), and a similar pattern was noted for immune cells (Fig. 7c)*.*

## Discussion

Accumulating evidence indicates that HCC is a disease of high genetic heterogeneity, which presents a major challenge for precision treatment [25, 26]. DRD1 functions as a determinant for G protein coupling between Gs heterotrimers in a complex with three catechol-based agonists [27], a noncatechol agonist, and a positive allosteric modulator for endogenous dopamine [27]. An increasing number of researchers have elucidated that DRD1 is a key player in several cancers [5, 9, 28] and a potential valid therapeutic target for certain receptors in cancer therapies [5, 17]. In this study, we characterized genomic heterogeneity, investigated transcriptional expression, uncovered genomic mutations and coexpressed genes, identified the hub gene network and prognostic implications, and established the tumour immune microenvironment landscape of multifocal HCC and its biomarker genes. By applying an online database, we demonstrated that DRD1 expression is significantly decreased across cancers at the transcriptional level. We identified remarkably attenuated DRD1 expression in HCC samples versus normal samples. Furthermore, we uncovered markedly mitigated DRD1 expression in advanced stages and tumour grades compared with early stages and grades of HCC. Previous studies have reported that DRD1 is abundantly expressed in human neural stem cells, suggesting that DRD1 has a role in the regulation of human brain development and associated neural system regulation [29]. We also identified that DRD1 expression may function in metabolic pathways such as ‘transport’, ‘metabolic process’, ‘oxidation–reduction process’, ‘extracellular exosome’, ‘transporter activity’, and ‘oxidoreductase activity’. In gastrointestinal stromal tumours (GISTs), researchers identified DRD1 as a key gene that was significantly expressed at low levels in imatinib-resistant GIST-48 and GIST-430 cells compared with GIST-T1 cells [30]. This work revealed DRD1 as a potential and promising target for overcoming imatinib chemotherapy resistance in GIST patients [30]. Another study showed that variants in DRD1 were relevant to the persistent and nonpersistent pain phenotypes in postsurgery breast cancer patients [31]. In glioblastoma, researchers observed that DRD1 was decreased in tumour tissues. In addition, DRD1 activation attenuated glioblastoma cell malignant activities through the mediation of autophagic activity [9]. Studies have also demonstrated that DRD1 activation is involved in dopamine’s cytotoxic effect on human neuroblastoma cells [32]. In the context of breast cancer bone marrow metastasis, a selective agonist of DRD1, A77636, suppressed breast cancer proliferation and migration [33]. Another study demonstrated the overexpression of DRD1 mRNA in a broad spectrum of common and rare cancers [34], and various signalling pathways were closely associated with tumour initiation and tumour progression [35, 36]. Dopaminergic signal transduction pathways have been characterized as inhibitory afferent input pathways in several studies [37, 38]. Furthermore, dopamine-mediated increases in DRD1 expression have been shown to suppress the proliferation and cytotoxicity of CD4 and CD8 + T lymphocytes in lung cancer patients in vitro [39]. It has been shown that the genetic profile of DRD1 may affect the activity of lymphocytes [40] and that the HCC microenvironment is important for both carcinogenesis and immunogenicity [41]. These studies indicated that DRD1 is involved in multiple immune-related regulations and may affect immune functions in disease and health control [42]. In this study, we evaluated the correlations between DRD1 expression in various cancers and immune cell infiltration/immune purity. Our findings indicated that DRD1 expression in HCC is not well correlated with immune purity. In addition, DRD1 expression has a significant correlation with macrophage, T cell and B cell infiltration, indicating that DRD1 expression might be involved in shaping the cancer immune microenvironment in HCC.

There are several limitations to our analysis. First, further clinical trials and more real clinical data are warranted to evaluate the application potential value of DRD1 in clinical practice. Second, the underlying regulatory mechanism of DRD1 in HCC needs to be further clarified and verified. Third, the clinical samples in this study were limited in number and diversity. We need to further assess more cancer types and clinical samples. In future studies, we need to collect more clinical samples and perform clinical experiments and bioinformatics analysis to improve and enhance our research impact.

## Conclusions

In this study, by adopting public databases, we comprehensively and systematically assessed DRD1 expression in HCC. Our findings demonstrated that DRD1 and its coexpressed genes are contributing factors in HCC initiation and proliferation-associated signalling pathways and the formation of an immune microenvironment. In conclusion, DRD1 is considered a favourable novel biomarker candidate for HCC prognosis evaluation.

## Supplementary Information

**Additional file 1: Figure S1.** Expression levels of hub genes in primary liver cancer and normal liver tissues. (a–e) The mRNA expression levels of AR, CAT, CYP3A4, ESR1 and SLC10A1 were remarkably upregulated in primary tumour tissues compared with normal tissues. (f, g). The mRNA expression levels of IRS1 and NS3C1 were significantly downregulated in primary tumour tissues. (h) The PIK3R1 mRNA expression levels showed no significant difference between tumour tissues and normal liver tissues.

**Additional file 2: Figure S2.** Association between hub gene expression and the prognosis of patients with HCC. (a) Kaplan–Meier analysis showed that patients with high AR expression had shorter OS times than those with low AR expression. (b–h) Kaplan–Meier analysis indicated that patients with increased CAT, CYP3A4, ESR1, SLC10A1, IRS1, NS3C1 and PIK3R1 expression had poorer OS.

## Data Availability

The datasets used and/or analysed during the current study are available from the corresponding author upon reasonable request.

## Abbreviations

HCC: Hepatocellular carcinoma
DRD1: Dopamine and dopamine receptor D1
LIHC: Liver Hepatocellular Carcinoma
TCGA: The Cancer Genome Atlas
TIMER: Tumour Immune Estimation Resource
GTP: Guanosine 5'-triphosphate
GEO: Gene Expression Omnibus
EGA: European Genome-phenome Archive
BLCA: Bladder urothelial carcinoma
BRCA: Breast invasive carcinoma
CESC: Cervical squamous cell carcinoma and endocervical adenocarcinoma
CHOL: Cholangiocarcinoma
COAD: Colon adenocarcinoma
GBM: Glioblastoma multiforme
HNSC: Head and neck squamous cell carcinoma
KICH: Kidney chromophobe
KIRC: Kidney renal papillary cell carcinoma
KIRP: Kidney renal papillary cell carcinoma
LUAD: Lung adenocarcinoma
LUSC: Lung squamous cell carcinoma
PCPG: Pheochromocytoma and paraganglioma
PRAD: Prostate adenocarcinoma
SKCM: Skin cutaneous melanoma
THCA: Thyroid carcinoma
UCEC: Uterine corpus endometrial carcinoma
PFS: Progression-free survival
OS: Overall survival
DSS: Disease-specific survival; gastrointestinal stromal tumour
