# Supplementary figures and images for: Dopamine and dopamine receptor D1 as a novel favourable biomarker for hepatocellular carcinoma
Source: Cancer Cell Int. 2021 Oct 30;21:586. doi: 10.1186/s12935-021-02298-9 (PMC8557590; doi:10.1186/s12935-021-02298-9)

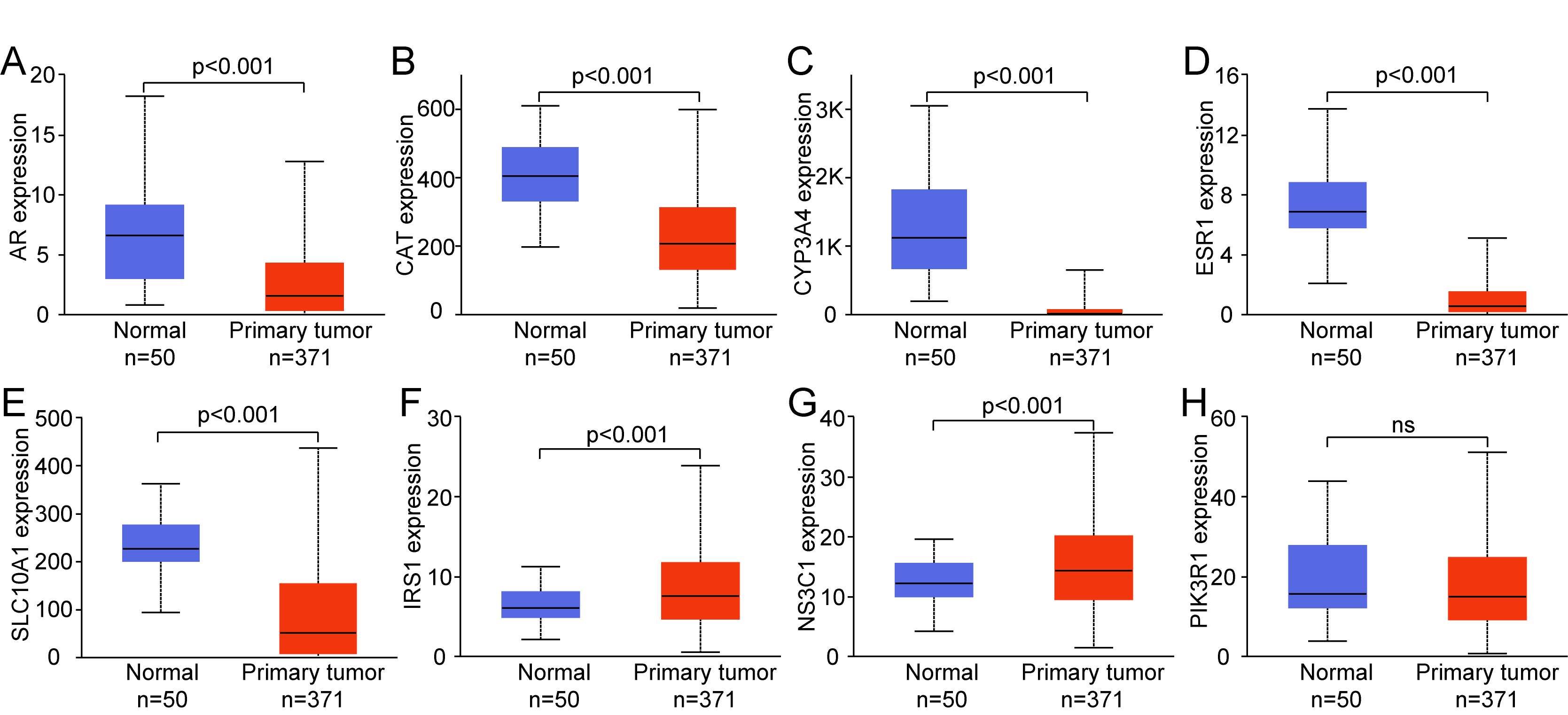

Supplement: Supplementary file 1 — Additional file 1: Figure S1. Expression levels of hub genes in primary liver cancer and normal liver tissues. (a–e) The mRNA expression levels of AR, CAT, CYP3A4, ESR1 and SLC10A1 were remarkably upregulated in primary tumour tissues compared with normal tissues. (f, g). The mRNA expression levels of IRS1 and NS3C1 were significantly downregulated in primary tumour tissues. (h) The PIK3R1 mRNA expression levels showed no significant difference between tumour tissues and normal liver tissues. [file 12935_2021_2298_MOESM1_ESM.tif]

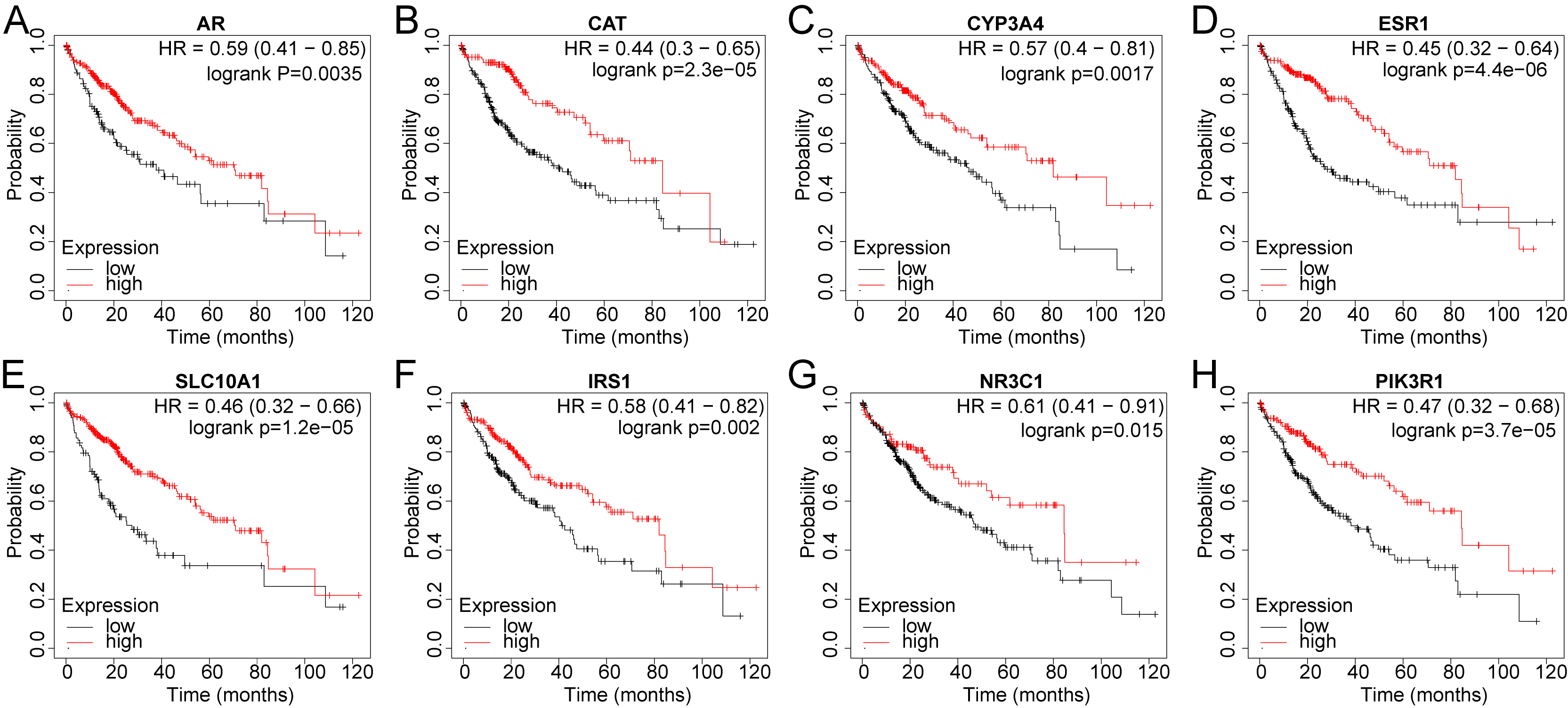

Supplement: Supplementary file 2 — Additional file 2: Figure S2. Association between hub gene expression and the prognosis of patients with HCC. (a) Kaplan–Meier analysis showed that patients with high AR expression had shorter OS times than those with low AR expression. (b–h) Kaplan–Meier analysis indicated that patients with increased CAT, CYP3A4, ESR1, SLC10A1, IRS1, NS3C1 and PIK3R1 expression had poorer OS. [file 12935_2021_2298_MOESM2_ESM.tif]
